# Genetic elimination of dopamine vesicular stocks in the nigrostriatal pathway replicates Parkinson’s disease motor symptoms without neuronal degeneration in adult mice

**DOI:** 10.1038/s41598-017-12810-9

**Published:** 2017-09-29

**Authors:** Elsa Isingrini, Chloé Guinaudie, Léa C Perret, Quentin Rainer, Luc Moquin, Alain Gratton, Bruno Giros

**Affiliations:** 1 Department of Psychiatry, Douglas Mental Health Research Center, McGill University, Montreal, Quebec, H4H 1R3 Canada; 2 Sorbonne Universités, Neuroscience Paris Seine, CNRS UMR 8246, INSERM U 1130, UPMC Univ Paris 06, UM119, 75005 Paris, France

## Abstract

The type 2 vesicular monoamine transporter (VMAT2), by regulating the storage of monoamines transmitters into synaptic vesicles, has a protective role against their cytoplasmic toxicity. Increasing evidence suggests that impairment of VMAT2 neuroprotection contributes to the pathogenesis of Parkinson’s disease (PD). Several transgenic VMAT2 mice models have been developed, however these models lack specificity regarding the monoaminergic system targeting. To circumvent this limitation, we created VMAT2-KO mice specific to the dopamine (DA) nigrostriatal pathway to analyze VMAT2’s involvement in DA depletion-induced motor features associated to PD and examine the relevance of DA toxicity in the pathogenesis of neurodegeneration. Adult VMAT2 floxed mice were injected in the substancia nigra (SN) with an adeno-associated virus (AAV) expressing the Cre-recombinase allowing VMAT2 removal in DA neurons of the nigrostriatal pathway solely. VMAT2 deletion in the SN induced both DA depletion exclusively in the dorsal striatum and motor dysfunction. At 16 weeks post-injection, motor symptoms were accompanied with a decreased in food and water consumption and weight loss. However, despite an accelerating death, degeneration of nigrostriatal neurons was not observed in this model during this time frame. This study highlights a non-cytotoxic role of DA in our genetic model of VMAT2 deletion exclusively in nigrostriatal neurons.

## INTRODUCTION

Parkinson’s disease (PD), is one of the most common neurodegenerative disorders affecting 1–2% of individuals older than 65 years old worldwide^1^. PD is a progressive disorder clinically characterized by a large number of motor features in particular rest tremor, bradykinesia, rigidity and loss of postural reflexes^2,3^. At the pathological level, processes leading to PD begin before the appearance of the typical motor symptoms, and by the time the disease is diagnosed about 70–80% of striatal dopamine (DA) is depleted and one third of substantia nigra (SN) DA neurons and striatal DA fibers are already lost^2,4–7^. Understanding the biochemical markers relevant to the pathogenesis of neurodegeneration in PD remain essential.

Oxidative stress contribution to PD neurodegeneration is currently well established^8^. Post-mortem studies have shown an imbalance between reactive oxygen species (ROS) production and the antioxidant protective system resulting in oxidative stress in the SN of PD patients^9,10^. Superoxide (O^2−^), hydrogen peroxide (H_2_O_2_), and the hydroxyl radical (OH∙), three important ROS in mammalian cells, can damage macromolecules including nucleic acids, lipids and proteins, leading to DA neuron degeneration, and neuron network dysfunction, ultimately progressing to PD^11^. For a better understanding of the oxidative stress mechanisms in PD, the MPTP (1-méthyl-4-phényl-1,2,3,6-tétrahydropyridine) model is currently used. MPTP’s metabolite MPP + , taken up by DA neurons inhibits the mitochondrial respiratory chain to induce ROS formation and elicits apoptotic neuronal death^8^. In both animals and humans, MPTP administration specifically induced dopaminergic neuronal loss and provokes parkinsonian motor symptoms^12–14^. One endogenous molecule thought to have similar cytotoxic properties to MPTP is DA itself. When free in the cytosol, DA auto-oxidation or its oxidation by the monoamine oxydase (MAO) results in ROS production, renders cells more vulnerable to other toxins^15–17^ and ultimately provokes apoptotic cell death^18,19^.

The type-2 vesicular monoamine transporter (VMAT2), a neuronal H^+^-ATPase antiporter, is of particular interest when studying DA neurodegeneration given its protective role from endogenous and exogenous toxicants^20,21^. The primary role of VMAT2 is to store monoamines into synaptic vesicles and to regulate stimulated monoamines quantal release^22,23^, resulting in protection of monoamines from cytoplasmic oxidation. Indeed, by regulating the amount of DA accumulated in the cytosol, VMAT2 protects cells from their own neurotransmitter toxicity^24^, suggesting that VMAT2 can modulate susceptibility of DA neurons to degeneration. Several different transgenic VMAT2 mice models have been developed to study the role of VMAT2 in monoaminergic signalling. The constitutive VMAT2 knockout (VMAT2-KO) mice, which die within few days after birth, displayed a 90–100% reduction in the total amount of monoamines in the entire brain confirming the impairment of monoamine storage and release induced by VMAT2 removal^25–27^. Moreover, the constitutive VMAT2 heterozygote (VMAT2-HET) mice, viable into adulthood with a 30–40% decrease in brain monoamines level, showed increase vulnerability of DA neurons to both MPTP and L-DOPA toxicity^26,28,29^. A recombinant event leading to the generation of a hypomorphic allele gave rise to VMAT2-knockdown (VMAT2-KD) mice which displays a 95% decrease in VMAT2 expression and function and have a 70%–90% decrease in brain monoamines. VMAT2-KD mice demonstrated an increase in DA- and MPTP-mediated toxicity that was sufficient to induce DA nigrostratial pathway neurodegeneration^29,30^. In human studies, VMAT2 gain of function haplotypes have been correlated with being protective for sporadic PD^31^. Moreover, DA uptake is reduced in PD patients suggesting that there may be an alteration of VMAT2 mediated vesicular filling in PD patients^32^.

Although current studies have outlined the relevance of VMAT2 in the understanding of PD pathogenesis neurodegeneration, VMAT2 deletion models lack specificity. Indeed, VMAT2-KO mice only survive for few days at birth^25,27^, therefore making it difficult to evaluate the long term behavioral and pathological outcomes of a defect in VMAT2 expression. Although VMAT2-HET and VMAT2-KD mice are viable into adulthood, the deletions of VMAT2 expression are unspecific to the DA system and to the SN, inducing some secondary effects not relevant to PD.

The purpose of the present study is thus to analyze VMAT2’s involvement in DA depletion-induced motor features associated with PD and examine the relevance of DA toxicity in the pathogenesis of neurodegeneration. We created viable VMAT2-KO mice specific to the DA nigrostriatal pathway; VMAT2 floxed engineered mice were stereotaxically injected in the SN with an adeno-associated virus (AAV) expressing the Cre-recombinase, allowing VMAT2 removal in DA neurons of the nigrostriatal pathway solely. In this model, DA depletion was observed exclusively in the dorsal striatum and was associated with motor deficits starting at 8 weeks post-injection ongoing until 16 and associated at this time with decreased food and water consumption, weight loss and accelerate death. However, during this time frame, these symptoms were not associated with any degeneration of nigrostriatal neurons. This study highlights a non-cytotoxic role of DA in our genetic model of VMAT2 deletion exclusively in nigrostriatal neurons, suggesting that preventing DA storage in vesicles and therefore DA release may not be responsible for the neurodegeneration seen in PD.

## Results

### Genetic, neurochemical and behavioral validation of specific ablation of the VMAT2 gene in the SN

Conditional ablation of the VMAT2 gene in the SN was obtained by injecting the AAV2 viral vector expressing the Cre recombinase, in the SN of 2 months old VMAT2^lox/lox^ mice. The Cre recombinase spliced out the VMAT2 floxed gene specifically in DA neurons of the SN.

**Figure 1:**
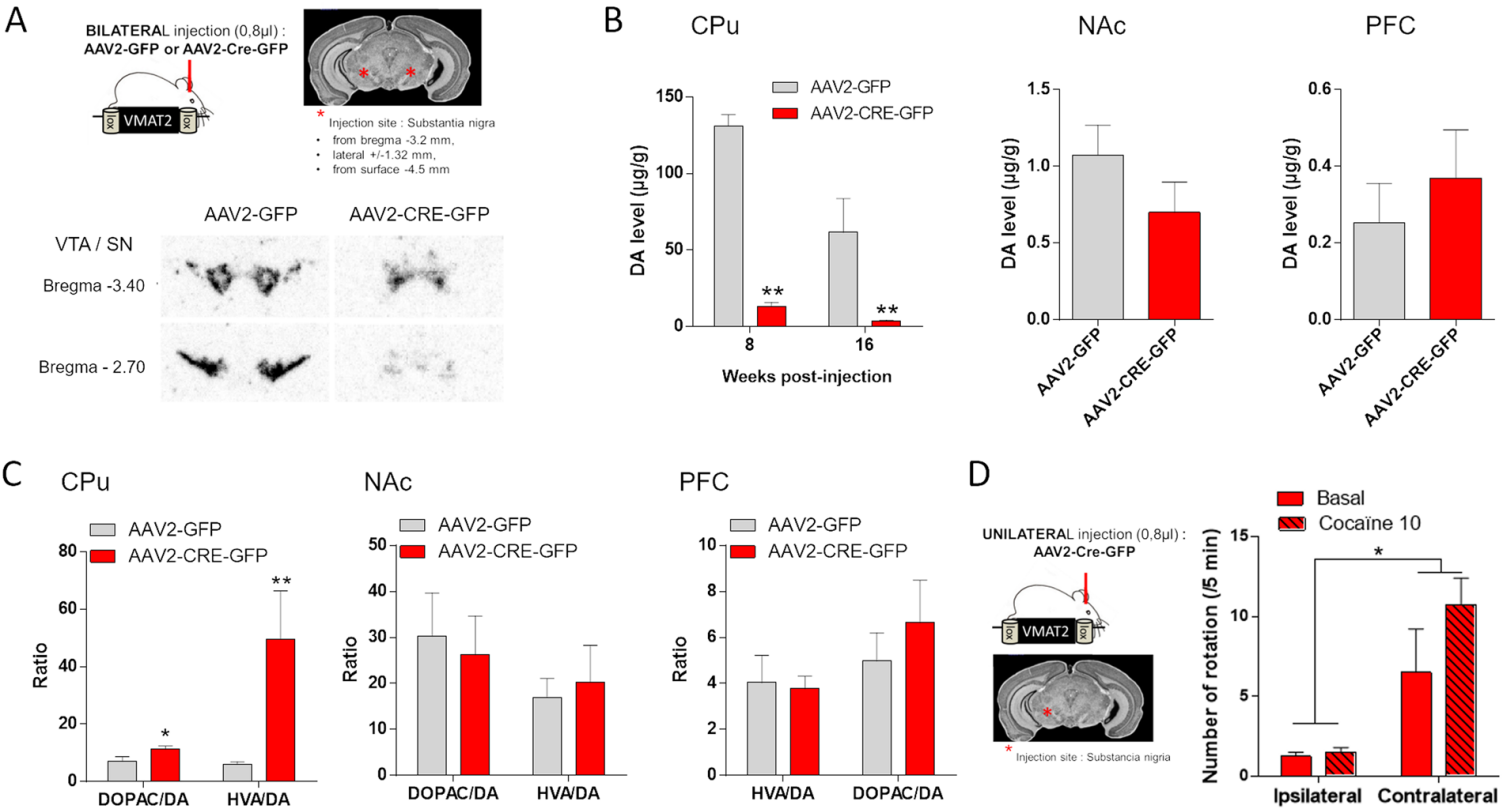
Genetic, neurochemical and behavioral validation of VMAT2 removal. (**A**) VMAT2 *in situ* hybridization in the SN and VTA of 2-month-old VMAT2^lox/lox^ mice injected bilaterally with AAV2-GFP or AAV2-CRE-GFP in the SN. (**B**) DA levels (µg/g of protein) measured by HPLC in the CPu, NAc and PFC of VMAT2^lox/lox^ bilaterally injected with AAV2-GFP (8 weeks: n = 5; 16 weeks: n = 6) or AAV2-CRE-GFP (8 weeks: n = 6; 16 weeks: n = 7) in the SN at 8 and 16 weeks’ post-injection (Mann-Whitney U: AAV2-GFP vs AAV2-CRE-GFP: 8 weeks: CPu **p < 0.0.01; 16 weeks: CPu **p < 0.01; NAc p = 0.22; PFC p = 0.43). (**C**) HVA/DA and DOPAC/DA ratio in the CPu, NAc and PFC at 16 weeks’ post-injection of VMAT2^lox/lox^ mice bilaterally injected with AAV2-GFP (n = 6) or AAV2-CRE-GFP (n = 7) in the SN (Mann-Whitney U: AAV2-GFP vs AAV2-CRE-GFP: HIAA/DA: CPu **p < 0.0.01; NAc - p = 0.28; PFC - p = 0.72. DOPAC/DA: CPu *p < 0.0.05; NAc - p = 0.62; PFC p = 0.83). (**D)** Number of ipsilateral and contralateral rotations over 5 minutes in both basal condition and 10 minutes post-cocaïne injection (10 mg/kg) in VMAT2^lox/lox^ mice AAV2-CRE uniterally injected (16 weeks’ post-injection, n = 4). (Wilcoxon test: ipsi vs contra: *p < 0.05). SN: Substancia nigra, VTA: Ventral Tegmental Area, CPu: Caudate Putamen, NAc: Nucleus Accumbens, PFC: PreFrontal Cortex, VMAT2: Vesicular Monoamine Transporter-2, AAV: Adenoassociated Virus, HVA: Homovanillic acid, DOPAC: 3,4-Dihydroxyphenylacetic acid.

To validate the specific conditional removal of VMAT2 in the SN, we assessed the efficiency of Cre-mediated splicing via radioactive VMAT2 *in situ* hybridization (Fig. 1A). A selective absence of VMAT2 mRNA labeling was observed in the SN starting at 8 weeks post-injection and ongoing, whereas VMAT2 mRNA was still expressed in DA neurons of the ventral tegmental area (VTA), demonstrating efficient and specific ablation of VMAT2 in the structure of interest.

The absence of VMAT2 mRNA observed in the SN of VMAT2^lox/lox^ injected with the AAV2 expressing the Cre recombinase was associated with a marked decrease in the tissue levels of DA in the dorsal striatum (Caudate Putamen, CPu), as measured via high performance liquid chromatography (HPLC) (Fig. 1B; Mann-Whitney U: AAV2-GFP *vs* AAV2-CRE-GFP: 8 weeks: U = 0, Z = 2.65, **p = 0.008; 16 weeks: U = 0, Z = 2.92, **p = 0.0034). In this same SN-projecting structure, the HVA:DA and DOPAC:DA ratios were increased in Cre-injected VMAT2^lox/lox^ mice compared with control mice (Fig. 1C; Mann-Whitney U: AAV2-GFP *vs* AAV2-CRE-GFP: HIAA/DA: U = 0, Z = −2.93, **p = 0.0034; DOPAC/DA: U = 6, Z = −2.07, *p = 0.038). This suggests that the amounts of DA was produced normally but quickly degraded due to the lack of VMAT2-dependent accumulation and protection in the vesicles. However, both DA level, HVA:DA and DOPAC:DA ratios were unchanged in the nucleus accumbens (NAc) and the prefrontal cortex (PFC), demonstrating DA nigrostriatal pathway specificity (Mann-Whitney U: AAV2-GFP *vs* AAV2-CRE-GFP: DA level: NAc - U = 12, Z = 1.21, p = 0.22; PFC - U = 15, Z = −0.78, p = 0.43; HVA/DA: NAc - U = 20, Z = 0.07, p = 0.94; PFC - U = 12, Z = −0.45, p = 0.65. DOPAC/DA: NAc - U = 17, Z = 0.5, p = 0.62; PFC - U = 14, Z = −0.091, p = 0.93).

After verifying specific ablation of VMAT2 in the SN associated with DA depletion in the dorsal striatum exclusively, we examined the consequences of unilateral DA depletion on rotational behavior. Unilateral injection of the cre-expressing viral vector in the SN of VMAT2^lox/lox^ mice led to contralateral rotations, an effect strengthened by 10 mg/kg cocaine injection (Fig. 1D; Wilcoxon test: ipsi vs contra T = 0.00, Z = 2.52; *p = 0.012).

### Behavioral consequences of DA depletion in the nigrostriatal pathway

**Figure 2:**
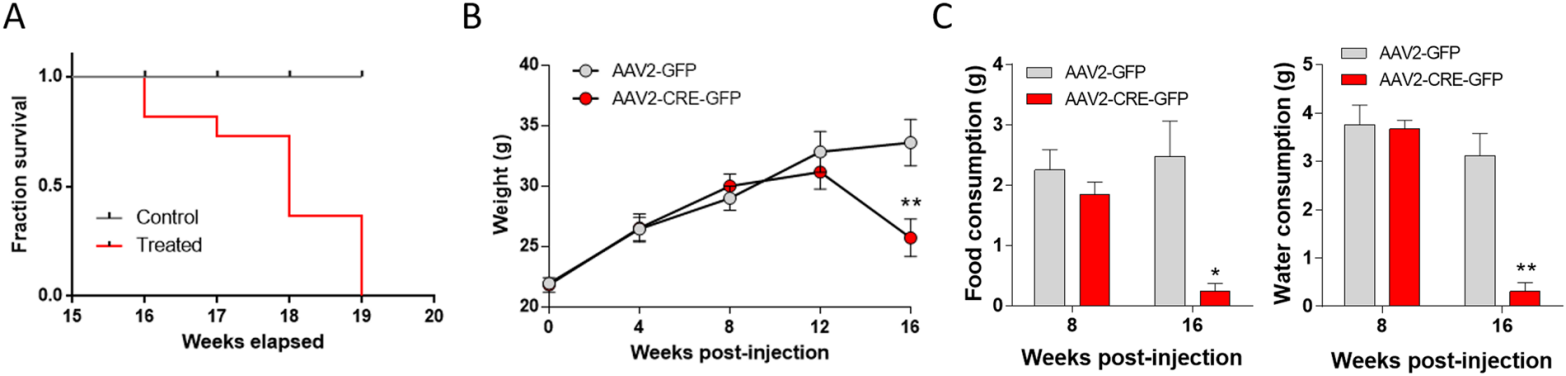
Survival, weight and food and water consumption. (**A**) Survival curve of VMAT2^lox/lox^ mice bilaterally injected with AAV2-GFP (n = 7) or AAV2-CRE-GFP (n = 8) in the SN (Log-rank (Mantel-Cox) test: p < 0.001). (**B**) Weight curve of VMAT2^lox/lox^ mice bilaterally injected with AAV2-GFP (n = 13) or AAV2-CRE-GFP (n = 11) in the SN (Mann-Whitney U: AAV2-GFP vs AAV2-CRE-GFP: Week 0: p = 0.91; Week 4: p = 0.91; Week 8: p = 0.88; Week 12: p = 0.43; Week 16: **p < 0.01). (**C**) Food and water consumption of VMAT2^lox/lox^ mice bilaterally injected with AAV2-GFP (n = 7) or AAV2-CRE-GFP (n = 7) in the SN at 8 and 16 weeks’ post-virus injection (Mann-Whitney U: AAV2-GFP vs AAV2-CRE-GFP: Food consumption: Week 8: p = 0.14; Week 16: *p < 0.05; Water consumption: Week 8: p = 0.53; Week 16: **p < 0.01). SN: Substancia nigra, VMAT2: Vesicular Monoamine Transporter-2, AAV: Adenoassociated Virus.

The survival curve of bilaterally Cre-injected VMAT2^lox/lox^ mice indicated a progressive decrease of the survival fraction of mice starting at 16 weeks post-injection with 100% of mice dead after 19 weeks whereas a survival rate of 100% was observed in control mice (Fig. 2A; Log-rank (Mantel-Cox) test: Chi square = 14,42, p = 0.0001). This effect on survival was associated with weight loss, at 16 weeks post-viral injection the weight of Cre-injected mice was 25.7 ± 1.5 g compared with 33.6 ± 1.9 for control mice (Fig. 2B; Mann-Whitney U: AAV2-GFP vs AAV2-CRE-GFP: Week 0: U = 69.5, Z = 0.12, p = 0.91; Week 4: U = 69.5, Z = −0.12, p = 0.91; Week 8: U = 69, Z = −0.14, p = 0.88; Week 12: U = 58, Z = 0.78, p = 0.43; Week 16: U = 19, Z = 3.04, p = 0.002). Food and water consumption of Cre-injected VMAT2^lox/lox^ mice compared with control was dramatically decreased at 16 weeks’ post-injection (Mann-Whitney U: AAV2-GFP vs AAV2-CRE-GFP: Food consumption: U = 6, Z = −2.14, *p = 0.032; Water consumption: U = 0.00, Z = −3.13, **p = 0.0017), explaining weight lost and ultimately death (Fig. 2C).

**Figure 3:**
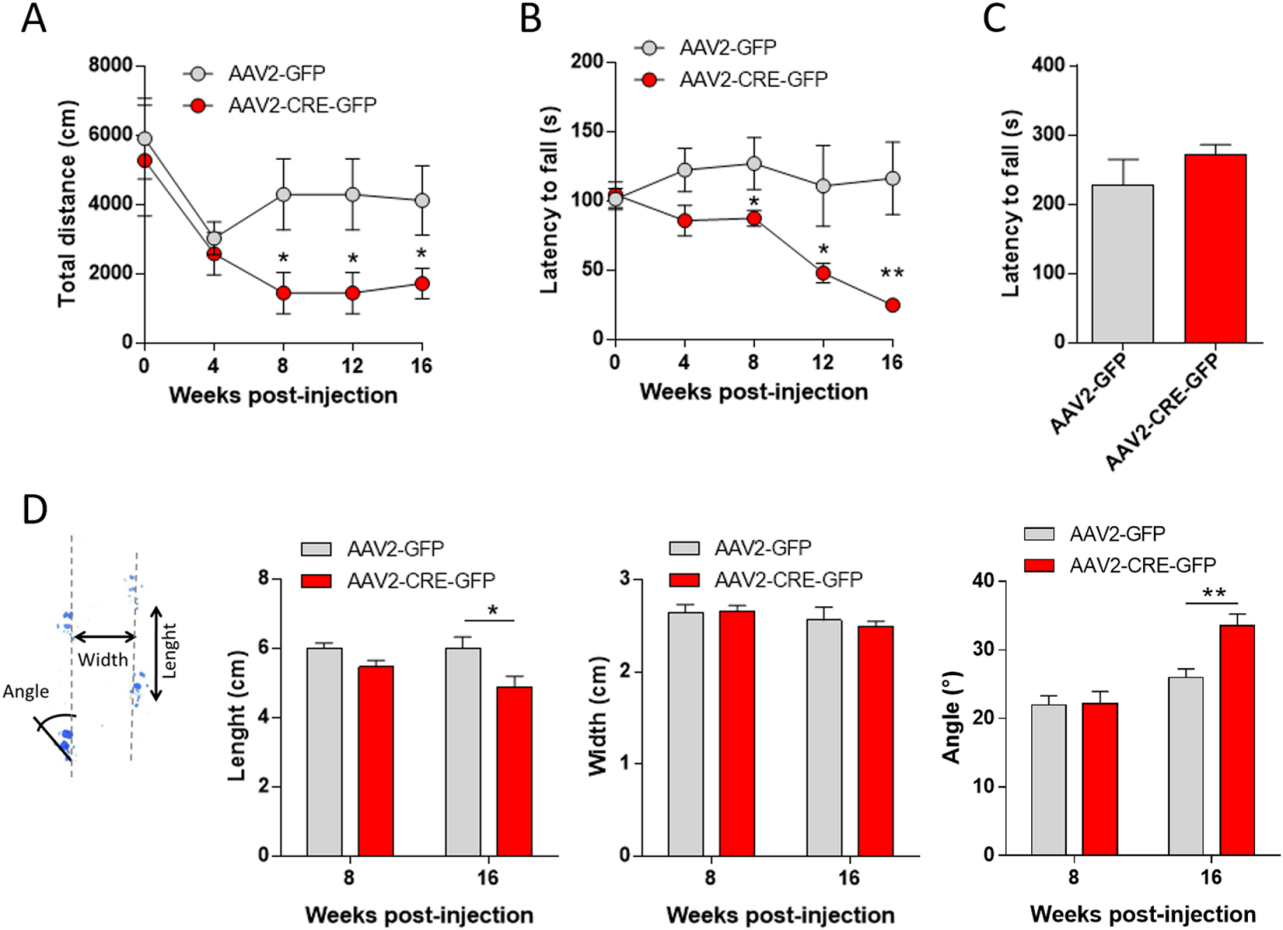
Motor behavioral consequences of nigrostriatal DA depletion. (**A**) Mean total distance (cm) measured at 5-min interval for 2 hrs every 4 weeks before and after AAV2-GFP or AAV2-CRE-GFP bilateral injection in VMAT2^lox/lox^ mice (n = 7 per groups; Mann-Whitney U: AAV2-GFP vs AAV2-CRE-GFP: week 8 to 16 *p < 0.05). (**B**) Mean latency to fall (s) in the accelerating rotarod over 4 consecutive sessions (4 to 25 rpm) every 4 weeks before and after AAV2-GFP (n = 8) or AAV2-CRE-GFP (n = 7) bilateral injection in VMAT2^lox/lox^ mice (Mann-Whitney U: AAV2-GFP vs AAV2-CRE-GFP: week 8 *p < 0.05; week 12 *p < 0.05; week 16 **p < 0.01). (**C**) Latency to fall (s) in the grip test 16 weeks’ post- bilateral injection of AAV2-GFP or AAV2-CRE-GFP in VMAT2lox/lox mice. Mann-Whitney U: AAV2-GFP *vs* AAV2-CRE-GFP: p = 0.73). (**D**) Gait analysis parameters in VMAT2^lox/lox^ mice 8 and 16 weeks’ post AAV2-GFP or AAV2-CRE-GFP bilateral injection. Stride length (cm), stance width (cm) and paw angle (°). No differences in gait at 8 weeks’ post-injection (Mann-Whitney U: AAV2-GFP *vs* AAV2-CRE-GFP: Length p = 0.11; width p = 0.90; Angle p = 0.93). Change the coordination of ambulation in AAV2-CRE-GFP injected mice compared to AAV2-GFP at 16 weeks’ post-injection (Mann-Whitney U: AAV2-GFP *vs* AAV2-CRE-GFP: Length *p < 0.05; width p = 0.75; Angle **p < 0.01). VMAT2: Vesicular Monoamine Transporter-2, AAV: Adenoassociated Virus.

Specific DA depletion in the nigrostriatal pathway induced motor ambulation and coordination alterations at 8 weeks’ post-viral injection ongoing. The mean total distance travelled in an open field (Fig. 3A, Mann-Whitney U: AAV2-GFP vs AAV2-CRE-GFP: week 8 - U = 6, Z = 2.37, p = 0.018; week 12 - U = 6, Z = 2.36, p = 0.018; week 16 - U = 9, Z = 1.98, p = 0.048) and the latency to fall in the rotarod test (Fig. 3B, Mann-Whitney U: AAV2-GFP vs AAV2-CRE-GFP: week 8 - U = 10, Z = 2.08, p = 0.037; week 12 - U = 9, Z = 2.20, p = 0.028; week 16 - U = 1, Z = 3.12, p = 0.0018) were decreased in Cre-injected mice compared to control from week 8 post-injection and this decrease continued up to week 16. Moreover, while the mean total distance travelled was significantly decreased at week 16 compared to week 0 in Cre- injected mice (Fig. 3A, Friedman test: Chi Sqr_(7,4)_ = 16.45, p = 0.0025; Wilcoxon test: Week 0 vs week 16 T = 1.00, Z = 2.19, *p = 0.028), this effect was absent in control mice (Fig. 3A, Friedman test: Chi Sqr_(7, 4)_ = 13.44, p = 0.0093; Wilcoxon test: Week 0 vs week 16 T = 7.00, Z = 1.18, p = 0.24). Similarly, in the rotarod accelerating test, a post-injection time effect was observed in the Cre-injected mice (Fig. 3B, Friedman test: Chi Sqr_(7,4)_ = 22.74, p = 0.00014) indicating a decrease in performance over-time while no time effect was observed in the control mice (Fig. 3B, Friedman test: Chi Sqr_(8,4)_ = 2.9, p = 0.57).

However, the latency to fall in the grip test at 16 weeks’ post-viral injection was unaltered in Cre-injected mice (Fig. 3C, Mann-Whitney U: AAV2-GFP *vs* AAV2-CRE-GFP: U = 25, Z = −0.35, p = 0.73). Finally, gait analysis indicated decreased in step length and angle whereas step width was unchanged, indicating change in the coordination of ambulation in AAV2-CRE-GFP injected mice compared to AAV2-GFP mice at 16 weeks’ post-injection (Fig. 3D, Mann-Whitney U: AAV2-GFP *vs* AAV2-CRE-GFP: Length U = 6, Z = −1.92, p = 0.054; width U = 16, Z = −0.32, p = 0.75; Angle U = 1, Z = 2.72, p = 0.0065) with no changes at 8 weeks (Mann-Whitney U: AAV2-GFP *vs* AAV2-CRE-GFP: Length U = 40, Z = −1.60, p = 0.11; width U = 64, Z = −0.12, p = 0.90; Angle U = 64, Z = −0.09, p = 0.93).

### Neuroanatomical consequences of DA depletion in the nigrostriatal pathway

To study whether DA depletion-induced motor alterations affected the neuroanatomical integrity of the nigrostriatal pathway, we looked at the expression of major DA markers.

**Figure 4:**
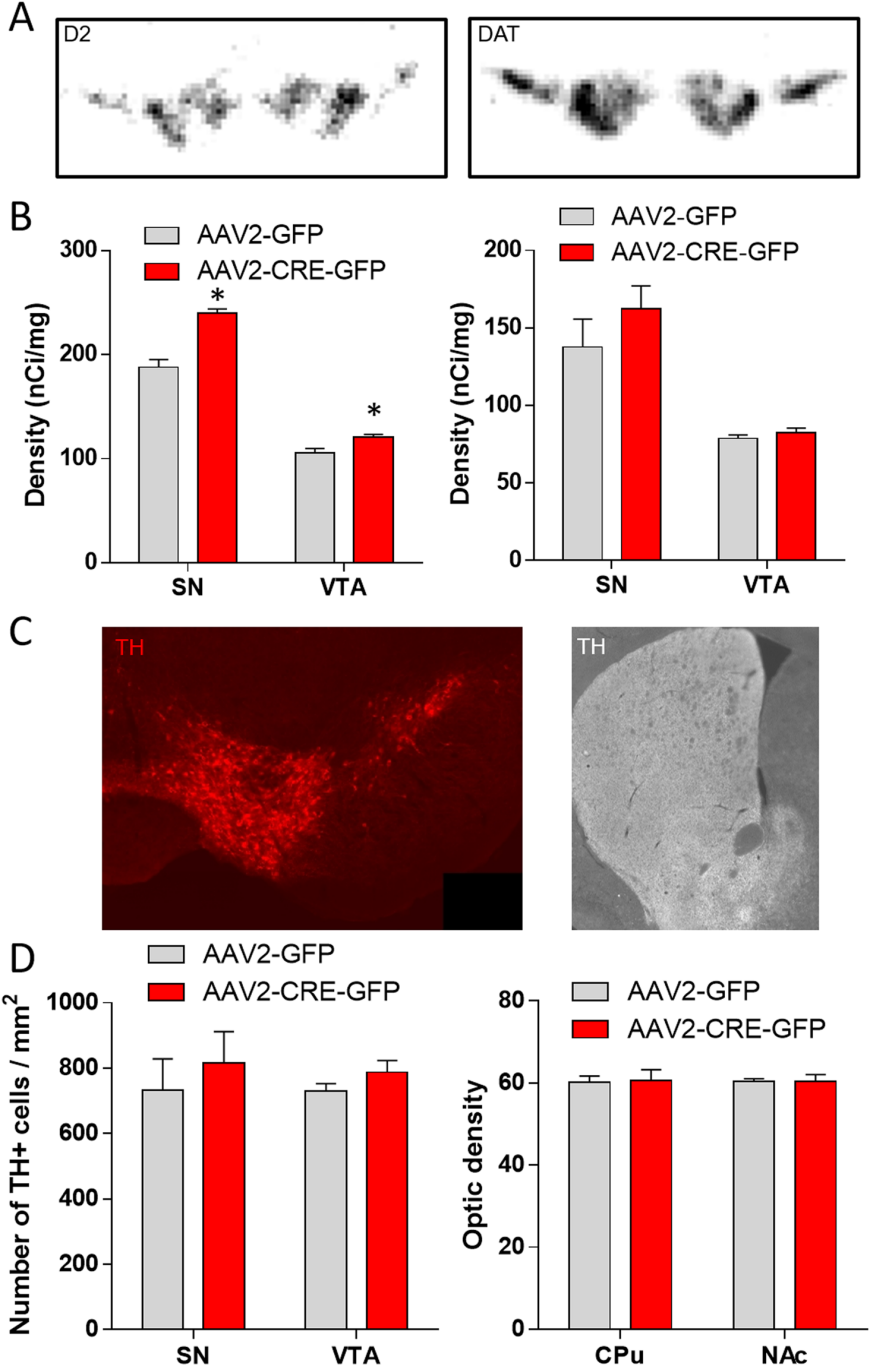
Neuroanatomical consequences of nigrostriatal DA depletion. (**A**) Illustration of D2 (left) and DAT (right) mRNA radioactive *in situ* labelling in the SN and the VTA. (**B**) Density (nCi/mg) of D2 (left) and DAT (right) mRNA expression in the SN and VTA, measured by radioactive *in situ* hybridization, 16-week after AAV2-GFP or AAV2-CRE-GFP bilateral injection in VMAT2^lox/lox^ mice (n = 4 per groups, Mann-Whitney U: AAV2-GFP *vs* AAV2-CRE-GFP: D2: SN *p < 0.05; VTA *p < 0.05; DAT: SN p = 0.47; VTA p = 0.14). (**C**) Illustration of TH immunostaining in DA neurons of the SN and the VTA (Left) and in the fibers in the CPu and NAc (Right). (**D**) Number of TH positive cell per mm2 in the SN and the VTA (Left) and optic density of TH positive fibers in the CPu and the NAc (Right) 16-week after AAV2-GFP or AAV2-CRE-GFP bilateral injection in VMAT2^lox/lox^ mice (n = 4 per groups, Mann-Whitney U: AAV2-GFP *vs* AAV2-CRE-GFP: TH + cells: SN p = 0.59; VTA p = 0.38; optic density: CPu p = 0.86; NAc p = 0.6). SN: Substancia nigra, VTA: Ventral Tegmental Area, CPu: Caudate Putamen, NAc: Nucleus Accumbens, PFC: PreFrontal Cortex, VMAT2: Vesicular Monoamine Transporter-2, AAV: Adenoassociated Virus, TH: Tyrosine hydroxylase.

DAT mRNA labeling measured by *in situ* hybridization were unchanged in the SN and the VTA of Cre-injected mice compared with controls (Fig. 4A, Mann-Whitney U: AAV2-GFP *vs* AAV2-CRE-GFP: SN – U = 5, Z = −0.72, p = 0.47; VTA – U = 8, Z = 0.14, p = 0.14), however, D2 mRNA labelling was increased in both the SN and the VTA of Cre-injected mice compared with control (Fig. 4B, Mann-Whitney U: AAV2-GFP *vs* AAV2-CRE-GFP: SN – U = 0, Z = −2.16, p = 0.03; VTA – U = 0, Z = −2.16, p = 0.03). Moreover, by performing TH immunostaining, we identified that the number of TH expressing neurons in the SN and in the VTA were identical between Cre-injected VMAT2^lox/lox^ mice and control mice (Fig. 4C,D, Mann-Whitney U: AAV2-GFP *vs* AAV2-CRE-GFP: SN – U = 4, Z = −0.53, p = 0.59; VTA – U = 3, Z = −0.88, p = 0.38). Finally, the density of TH positive fibers in both the CPu and the NAc was similar between both genotypes (Fig. 4C,D, Mann-Whitney U: AAV2-GFP *vs* AAV2-CRE-GFP: CPu – U = 5, Z = 0.18, p = 0.86; NAc – U = 4, Z = 0.53, p = 0.6). These results indicate that in our model of DA depletion with induced motor alterations, DA cytoplasmic accumulation due to the lack of VMAT2 expression is not sufficient to induced neurodegeneration.

## Discussion

The present study directly addresses a role for the specific deficit of DA vesicular storage in the nigrostriatal pathway in the etiology of neuronal cell death in neurodegenerative disorders, and most particularly Parkinson disease. To reach this objective, we used a unique genetic model to remove in adult mice VMAT2 expression in DA neurons of the SN specifically. In conditional VMAT2 KO mice model, we previously showed that early somatic deletion of VMAT2 exclusively in DA neurons causes a rapid postnatal death^33^, whereas the specific genetic deletion of VMAT2 in noradrenergic or serotonergic neurons does not prevent mice to live and grow up to adulthood^33–35^. This early postnatal death observed in conditional DA neurons-specific VMAT2 KO mice was similar to the one observed in constitutive VMAT2^25–27^ or DA-specific TH^36^ KO mice. Therefore, to circumvent this limitation, we used floxed VMAT2 mice, that have no phenotypical alterations by themselves and we initiated the VMAT2 gene splicing by stereotaxic injection of a virus expressing the Cre-recombinase in adult mice.

It is well known that unilateral DA depletion induces rotational behavior, however the direction of the rotation is dependent upon the model or the drugs used. Contralateral rotational behavior is exhibited by 6-OHDA-lesioned rats, whereas ipsilateral rotations are produced in animals with electrolytic lesions^37^. Moreover, in the 6OHDA model, while amphetamine induced ipsilateral rotation, contralateral rotation was observed with apomorphine^38^. It has also been shown that activation of the direct pathway (D1) induced contralateral rotation in contrast to activation of the indirect pathway (D2) which induced ipsilateral rotation^39^. In our model of genetic removal of VMAT2, unilateral injection of the Cre-expressing AAV2 in the SN shows the expected behavioral consequence of DA transmission imbalance; the contralateral rotations are of the same magnitude as those seen in unilateral 6-OHDA lesioned rats^37,40,41^, and these spontaneous rotations are increased upon cocaine administration. Interestingly, despite that VMAT2 mRNA signal disappeared as early as 8 weeks after bilateral injection, we did not reliably observed these spontaneous rotations before 16 weeks following the viral administration. Accordingly, at 8 weeks following the bi-sided virus injections, DA levels are already significantly decreased by 90%, and the mice show significant locomotion and coordination deficits, but have a normal food and water intake and no weight loss. 16 weeks following viral administration in the SN, VMAT2^lox/lox^ AAV2cre injected mice showed a total collapse of DA tissue-concentration in the striatal target region, but not in the ventral striatum nor frontal cortex that are mostly innervated by DA fibers originating from the VTA^42^. At this time, when DA levels are as low as only 4–5% of the control, mice stop eating and drinking and all died within the next two weeks. We hypothesizes that the manifestation of contralateral rotational behavior, at 16 weeks post-virus injection, may suggest that the <5% remaining DA preferentially activate the direct pathway, as a consequence of DA higher affinity for the D1 DA receptor^43^. Moreover, activation of the direct DA pathway is known to increase ambulation in mice^39^. Acting preferentially on this pathway could be a compensatory mechanism to counteract the induced motor dysfunction. The absence of vesicular DA release is not paralleled by any anatomical alterations, as assessed by the key DA markers: the dopamine transporter (DAT), the D2 dopamine receptor and the tyrosine hydroxylase (TH) enzyme in cell-bodies and terminals. In contrast, an increase in D2 dopamine receptor mRNA is observed in the SN and the VTA of DA-depleted mice. This is in agreement with the absence of a role of DA in the development and maintenance of DA circuitry that have been observed in TH KO mice^36,44^.

Vesicular transporters are mostly localized in nerve terminals, at distance from the cell body from where they have to be relocated, and this may advocate that they should have a quite robust maintenance giving their essential role in transmission^45–47^. However, at the present time and to our knowledge, we have absolutely no information regarding their half-life time, for any of them. Since the VMAT2-KD mice, expressing only 5% of VMAT2^30,48^, survive for several months, we can infer that after 16 weeks, our dying VMAT2^lox/lox^-cre mice must be below this 5% threshold of VMAT2 expression. Considering that it would take 5 half-life periods to reach 3.125% of expression and 6 half-time periods to reach 1.56%, and that mRNA depletion is reached in one week, it would imply that one half-life time period is comprised between 17.5 (15 Wk/6) and 21 (15 Wk/5) days. This value, that represents the first attempt to evaluate the life cycle of these transmembrane vesicular transporters, clearly indicates a very slow turnover rate and metabolism of this vesicular protein. Further experimental data, using complementary approaches, should now confirm this first report.

To evaluate a cytotoxic role for cytoplasmic DA, we tested the idea that the absence of DA-vesicular storage and the sequential increase of cytoplasmic DA may possibly trigger cell death through mitochondrial dysfunction and ROS generation^49–52^. Supporting this hypothesis, a decrease of VMAT levels in Parkinson brains have been substantially found^53–55^. However, our findings would indicate that it is more likely a consequence of DA neurons death, rather than a cause for their degeneration. We observed that, compared to 6-OHDA or MPTP lesions of the DA nuclei^12–14^ which induce a cellular death of DA neurons, the genetic deletion of VMAT2 we engineered here does not affect the anatomy of the DA system after 16 weeks, as observed by the absence of alteration in presynaptic markers of the dopaminergic system. The dopamine transporter (DAT) regulates extracellular DA level through reuptake of the release transmitter into presynaptic DA neurons. Tyrosine Hydroxylase (TH) is the rate-limiting enzyme in DA biosynthesis and its expression constitute a specific indicator of DA production^56,57^. The D2 presynaptic auto-receptor regulates DA transmission by inhibiting the probability of vesicular DA release^58^, decreasing DA synthesis^59^ and altering the uptake of DA^60^. Accordingly, DAT, the D2 presynaptic receptor and TH are the most appropriate markers of damage to the striatal DA terminals in PD^61,62^. In our model, we did not find any alterations in DAT mRNA expression in the SN and TH immunoreactivity in both the striatum and the SN. These results are partially in accordance with the one observed in reserpine treated mice where no effect was found on DAT immunoreactivity but a decrease in striatum TH immunoreactivity was observed^63^. However, transgenic mice expressing only 5% of VMAT2 (VMAT2-KD mice) present an age-dependent degeneration of nigrostriatal dopamine neurons. In this model of unspecific disruption of DA storage, mice exhibit decreased DAT and TH immunoreactivity in the striatum associated with increased oxidative damage^30^. Finally, in our model of dopamine depletion, we find an increased expression of the D2 dopamine receptor mRNA in the SN and the VTA. In accordance with this effect, it was found that in mice lacking the dopamine transporter DAT, which display biochemical and behavioral dopaminergic hyperactivity, the D2 autoreceptors mRNA, measured by *in situ* hybridization is reduced in the ventral midbrain^64,65^. In DAT-KO mice, this decrease was found mice to counteract the increased DA neurotransmission. In our experimental condition, we hypothesize that in absence of DA transmission, increased D2 autoreceptor expression may compensate the dramatic change in DA homeostasis.

Genetic and pharmacological reduction of VMAT2 resulted in lower tissue level of striatal DA. Consistently, in DA terminals of the striatum originating from the SN, we observed a dramatic collapse of DA concentration. As observed in VMAT2-KD mice, this reduction is accompanied by an increase in the ratios of DA metabolites to DA (HVA/DA and DOPAC/DA) suggesting an increase in DA turnover. This highlights that the genetically engineered deficit of DA storage, rather than uprising the accumulation of cytoplasmic DA, is accelerating the metabolic outcome of DA.

What is the actual value of animal models to assess the molecular and cellular etiology of PD? One good illustration of these timescale is by comparing the genetic deletion of the DAT in human and mice. In humans, mutations of the DAT gene is responsible for a very severe early onset of PD, which first motor symptoms usually appeared during the first postnatal year and young children are dying before the age of 10 years^66–68^. In mice, the knockout of DAT does not trigger such a dramatic phenotype^64^, even though it has been reported a sporadic death of about one third of DATKO mice, usually between the 20^th^ and 50^th^ postnatal week^69^ when the mice where on a C57BL/6 background, whereas such high proportion of death is not seen when the DATKO mice are on a C57BL/6xDBAD/2 hybrid background^70^. Actually, beside the neurotoxin-induced animal models, where DA neuronal death occurred quite rapidly, none of the genetic models of PD, based on gene invalidation or mutation, show any degeneration of DA neurons^71^. In our VMAT2 KO mice targeted to the SN, death occurred within 4 to 5 months, whereas in human, it takes decades for the catecholamines neurons to die upon the reaching of motor symptoms.

In summary, we engineered a new transgenic mice model of VMAT2 removal-induced DA depletion specifically in the nigrostriatal pathway. DA homeostasis alteration induced in this model reproduce motor deficit observed in PD, however, it is not sufficient to reveal DA cell loss and neurodegeneration characterizing PD physiopathology. Although this model of DA depletion does not fully recapitulate the complexity of the human disease, it constitutes the first model of dissociating DA depletion pathway. Here, we are able to target exclusively the nigrostriatal pathway without affecting the mesocorticolimbic pathway. This innovative model could help figure out the specific involvement of these two distinct DA pathways in both motor and non-motor function and dysfunction.

## Methods

### Animals

Animal housing, breeding, and care were operated in accordance to the Canadian Council on Animal Care guidelines (CCAC; http://ccac.ca/en_/standards/guidelines) and all methods were approved by the Animal Care Committee from the Douglas Institute Research Center under the protocol number 5570. All methods were performed in accordance with the relevant guidelines and regulations.

The mice were kept under standard conditions at 22 ± 1 °C, a 60% relative humidity, and a 12-h light-dark cycle with food and water available *ad libitum*.

The floxed VMAT2 mouse strain were obtained from the Mouse Clinical Institute (Institut Clinique de la Souris, MCI/ICS, Illkirch, France). Heterozygous VMAT2 floxed mice (VMAT2^lox/+^) were crossed to generate homozygote mice (VMAT^lox/lox^) necessary for Cre-expressing viral vector injection. VMAT2^lox/lox^ mice were maintained on a C57BL/6 J background. After weaning and sexing, mice were housed in group of 4–5 animals per cage. Male VMAT^lox/lox^ mice were used for stereotaxic surgery at 2 months of age.

### Stereotaxic surgery

Mice VMAT^lox/lox^ were anesthetized with isoflurane gas combined with oxygen, and placed onto the stereotaxic apparatus. Once the skull was exposed and a hole drilled bilaterally, the recombinant AAV2.CMV.HI.eGFP-Cre.WPRE.SV40 or control AAV2.CMV.PI.eGFP.WPRE.bGH virus vectors (Penn Vector Core, AV-2-PV2004 and AV-2-PV0101) were injected at a 0° angle into the SN (Anteroposterior from bregma: −3.2; Lateral from the midline: ± 1.32; Dorsoventral from the surface: −4.5). VMAT^lox/lox^ were randomly assigned to viral injection. Virus injections (0.5 µl per side at a rate of 0.05 μl/min) were done through an internal cannula connected via tubing to a 10-μl Hamilton micro syringes mounted on a micro-drive pump (WPI, Sarasota, FL, USA).

### Quantitative *in situ* hybridization

At 8-week or 16-week post-stereotaxic injection, mice brains were collected after decapitation and frozen in isopentane at −30 °C. Brains were sliced in coronal sections (10 μm thick) using cryostat (Leica CM3050S) and rinsed in 0.1 M PBS, SSC 10 M and treated with 0.25% ethanol. [^35^S]-dATP oligonucleotides (**VMAT2**: 5′-GAG GAA CAC GAT GAA CAG GAT CAG CTT GCG CGA GT-3′; 5′-CTA CGA CGG TGA GCA GCA TGT TGT CTA GCA GCA G-3′; **D2:** 5′-GTA GTT GTA GTG GGG CCT GTC TGG CTT CCC TTC G-3′; 5′-GGA CTG TCA GGG TTG CTA TGT AGA CCG TGG TGG G-3′; 5′-GTG AGC TGG TGG TGA CTG GGA GGG ATG GGG CTA TA-3′; 5′-GAA GGC GCT GTA GAG GACT GGT GGG ATG TTG CAG-3′; 5′-ACT CAG CAG TGC AGG ATC TTC ATG AAG GCC TTGC-3′; 5′-TCC TTC TGC TGG GAG AGC TTC CTG CGG CTC ATC GT-3′; 5′-GAT GAT AAA GAT GAG GAG GGT GAG CAG CAT GGC A-3′; **DAT**: 5′-GAC TTC CTG GGG TCT TCG TCT CTG CTC CCT CTA C-3′;5′-GTA GGC CAG TTT CTC TCG AAA GGA CCC AGG CAG G-3′; 5′-GGT ATG CTC TGA TGC CGT CTA TGG CTC CAG GGA G-3′; 5′-GCC TGA GTG GCA GTA GCC TGA GCT GGT TTC AAG G-3′; 5′-GTT GGC CCA GTC GGG GAA GAT GTA GGC TCC GTA GT-3′) were synthesized with terminal transferase (Amersham, Biosciences) to obtain a specific activity of 5 × 10^−8^ dpm/µg. Sections were covered with 70 µl of hybridization mix and 5 × 10^−5^ dpm of each labeled oligonucleotide, and incubated overnight at 42 °C in a humid chamber. Following washes and dehydration, slides were air-dried and exposed to a BAS-SR Fujifilm Imaging Plate for 5 days. The plates were scanned with a Fujifilm BioImaging Analyzer BAS-5000. Regions identification was based on Franklin and Paxinos Mouse Atlas^22^.

### High Performance Liquid Chromatography

HPLC was performed on micropunches of 1 mm diameter from the dorsal striatum (CpU), the nucleus accumbens (NAc), and the prefrontal cortex (PFC) of VMAT^lox/lox^ at 8 and 16 weeks post viral injection. After decapitation, brains were collected, frozen in isopentane at −30 °C and stored at −80 °C. Micropunches of specific structures were homogenized in a solution containing 45 μl of 0.25 M perchlorate and 15 μl of DHBA (100 mg/ml) which served as an internal standard. Following centrifugation at 10,000 rpm for 15 minutes at 4 °C, the supernatant was isolated to detect DA, dihydroxyphenylacetic acid (DOPAC), homovanillic acid (HVA), NE, serotonin (5-HT), and 5-hydroxyindolacetic acid (HIAA) using high pressure liquid chromatography with electrochemical detection (HPLC-EC). Samples were run through a Luna C18 (2) 75 × 4.6 mm 3 μm analytical column at a flow rate of 1.5 ml/min and the electrochemical detector (ESA Coularray, model #5600 A) was set at a potential of −250 mV and +300 mV. The mobile phase consisted of 6% methanol, 0.341 mM 1-octanesulfoic acid sodium salt, 168.2 mM sodium acetate, 66.6 mM citric acid monohydrate, 0.025 mM ethyenediamine-tetra-acetic acid disodium (EDTA) and 0.71 mM triethylamine adjusted to pH 4.0–4.1 with acetic acid. Using ESA’s CoulArray software, the position of the peaks for each metabolite was compared to an external standard solution containing 25 ng/ml DHBA, DA, NE, 5-HT, DOPAC, HVA, and 50 mM acetic acid. In parallel, pellets were reconstituted in 50 μl of 0.1 N NaOH and kept for protein quantification using a BCA^TM^ Protein Assay Kit (Fisher Scientific, Ontario, Canada). Each analyzed sample was measured in µg/g of protein.

### Immunofluorescence labeling

Mice were perfused with 0.9% NaCl followed by 4% paraformaldehyde (PFA). Brains were collected and post-fixed in 4% PFA for 2 hours, and kept in a 15% sucrose solution at 4 °C before being sliced in coronal sections (40 μm thick) using a cryostat (Leica CM3050S). Free-floating slices of VMAT^lox/lox^ mice injected with the AAV2-GFP or the AAV2-CRE-GFP into the SN were rinsed in PBS 0.1 M and incubated overnight with a rabbit anti-TH (1/4000; Santa Cruz, sc-14007) primary antibody diluted in PBS 0.1 M, 2% normal goat serum (NGS), and 0.3% triton. After washes, slices were incubated for 2 hours in secondary antibody, a goat anti-rabbit alexa 555 (1/500; Life technologies, A-21429) diluted in PBS 0.1 M with 2% NGS, and 0.3% triton. Sections were then rinsed and mounted onto gelatin-coated slides under Vectashield mounting medium.

The number of TH expressing cells in the SN and the VTA was counted bilaterally on every three sections (total of 6 sections per animal) throughout the entire nucleus from bregma −2.70 to bregma −3.88. The density of TH positive fibers was analyzed bilaterally on every three slices for a total of seven sections per animal throughout the NAc from bregma −1.70 to bregma 0.74 and on nine sections throughout the CPu from bregma −1.70 to bregma 0.14.

### Spontaneous and evoked rotations

At 16 weeks’ post unilateral viral injection in the SN, VMAT2^lox/lox^ mice were placed in a cylindric open field (40 cm diameter). Ipsilateral and contralateral rotation, defined as each 360° rotations that contain no turn of more than 90° in the opposite direction, were recorded during 5 minutes in baseline conditions and after cocaine injection (10 mg/kg, recording started 10 minutes after injections). Cocaine hydrochloride (Sigma Aldrich) diluted in a NaCl 0.9% was administrated intraperitoneally.

### Locomotor activity and motor coordination

Spontaneous locomotion was measured before the viral injection and every 4 weeks after the viral injection in an Omnitech digiscan activity monitor. Plexiglas open-field chambers (40 cm^2^) with photocells placed on bottom and lateral surfaces allowed to measure the total distance travelled at 5-minute intervals for 2 hours.

Motor coordination was assessed by an accelerating rotarod (ROTO-ROD, Series 8, IITC Life Sciences) before the viral injections and every 4 weeks after the viral injection. The mice latency to fall was recorded over 4 consecutives trials at a rotating speed range from 4 to 25 accelerating rpm for a maximum of 5 minutes.

### Gait analysis

At 8 and 16 weeks’ post-viral injection, the gait of VAMT2^lox/lox^ mouse during spontaneous walk/trot locomotion was analyzed to identify specific paw step (stride length; stride width, and stride angle).

### Grip test

16 weeks post viral injection, VMAT^lox/lox^ mice were placed in the center of the wire mesh screen (12 mm squares of 1 mm diameter wir) and the screen were rotated to an inverted position over 2 sec, with the mouse’s head declining first. The screen was held steadily 40–50 cm above a padded surface. The time when the mouse falls off was recorded, or it was removed when the criterion time of 300 sec was reached.

### Statistical analysis

The results are expressed as mean ± SEM (standard error of the mean). No statistical methods were used to pre-determine sample sizes, but our sample sizes were similar to those generally employed in literature for the same paradigms. Statistical analyses were performed using Statistica software. Since the sample sizes were small (*n* < 30) and/or the variables did not follow a normal distribution (Shapiro–Wilk test) and/or the variances were not equal among groups (Leven test), we used a nonparametric statistical analysis. For 2 × 2 comparisons, we performed the Mann–Whitney *U* test for two independent samples (AAV2-GFP *vs* AAV2-CRE-GFP) and the Wilcoxon matched pairs test for dependent samples (8 weeks *vs* 16 weeks; Ipsilateral *vs* contralateral). For multiple repeated measures analysis (Total distance travelled and latency to fall in the rotarod), we used the Friedman test followed by the Wilcoxon test for the 2 × 2 comparisons. The comparison of the survival distribution between AVV2-GFP and AAV2-CRE-GFP groups was analyzed using the log-rank (Mantel-cox) test. Optic density was quantified using MCID for VMAT2, D2 and DAT mRNA labelling and the number of TH positive fibres in the striatum (CPu *vs* NAC) with image J. A P value < 0.05 was taken to indicate statistical significant differences between groups.
